# Quantitative musculoskeletal monitoring and analysis in aquatic rehabilitation

**DOI:** 10.3389/felec.2025.1566899

**Published:** 2025-04-02

**Authors:** Abu Bony Amin, Ebenezer Asabre, Sina Razaghi, Yeonsik Noh

**Affiliations:** 1 Department of Electrical and Computer Engineering, University of Massachusetts Amherst, Amherst, MA, United States; 2 Elaine Marieb College of Nursing, University of Massachusetts Amherst, Amherst, MA, United States

**Keywords:** aquatic rehabilitation, bicep curls, tricep kickbacks, musculoskeletal monitoring, quantitative analysis, waterproof wearable device

## Abstract

The benefits of aquatic rehabilitation have been demonstrated to promote wellbeing and facilitate motor recovery in middle-aged adults and geriatrics. Individualized patient-centered treatment is essential to accelerate and improve the rehabilitation process of neurological and orthopedic patients. Although aquatic therapy and rehabilitation are well known to be beneficial to these populations, it can be challenging for therapists to visualize and monitor patient progress and provide individualized feedback to ensure correct movement as planned. To establish the suitability of the developed wearable device in an aquatic environment, this study compared the extracted features of the sEMG and IMU data in on-land and aquatic environments for the bicep curls (BC) and tricep kickback (TK) protocols. We conducted a systematic analysis of the reproducibility and precision of the sEMG-IMU characteristics to assess the feasibility of the device for practical applications. While time and frequency domain features of sEMG were higher in aquatic environments compared to on-land, the Intraclass Correlation Coefficient (ICC) for these features ranged from 0.81 to 0.98, and the Coefficient of Variation (*CV*%) exhibited a range of 5.7% to 14.4%, highlighting reproducibility and correlation across environments in the two protocols. Environment. Moreover, for frequency domain the reproducibility and precision of the sEMG recordings for each muscle in this study were obtained high (*ICC* = 0.92 – 0.96, *CV*% = 5.4 – 13.8%). It’s noticeable that the observed acceleration data is almost similar to the same movement was maintained throughout the exercise. Eventually, the quantitative result is used to cluster the protocol types along with various repetitions to promote the personalized aquatic rehabilitation.

## 1 Introduction

Aquatic therapy also known as hydro-kinesitherapy, with its utilization of water’s unique physical properties such as buoyancy, hydrostatic pressure, and thermodynamics, holds promise as an effective approach for motor rehabilitation in individuals with neuromuscular diseases or injuries (Marta et al., 2020; Li et al., 2017; Silvers and Dolny, 2011; Iliescu et al., 2020). This therapeutic modality not only enhances motor recovery and wellbeing but also offers a safe and comfortable environment for rehabilitation, accommodating varying levels of function and capacity. Additionally, aquatic rehabilitation, particularly during the initial phases of musculoskeletal movement, take advantage of water’s properties to induce distinct physiological and biomechanical responses, including the alteration of resistance through drag force (Kaneda et al., 2013). This suggests that aquatic therapy can play a valuable role in improving motor function and overall rehabilitation outcomes for patients with neurological disorders and motor impairments (Marta et al., 2020; Iliescu et al., 2020). It is inferable that water can be used as a “rehabilitation tool” by combining the benefits of therapeutic exercise and immersion.

Despite the increasing popularity and efficacy of aquatic exercise, there is still a lack of evidence in terms of quantitative measurement and analysis for the movements during aquatic therapy and rehabilitation. In the past, underwater activity measurements were predominantly investigated to monitor swimming performance. The conventional method for quantifying underwater movements entails video-based systems, which require cameras both above and below the water (Komar et al., 2012). However, because this method is burdensome, time-consuming, and restricted to specialized instrumented pools, it is impractical for use in a broader range of rehabilitation settings (Mooney et al., 2015) and even not direct measurement of aquatic activities. In addition, the data analysis depends on computationally intensive computer vision algorithms, making it less accessible to non-technical users. Besides, in most cases when therapists work on aquatic therapy and rehabilitation for patients, it is difficult for them to make accurate assessments of the effectiveness of patient movements in their rehabilitation. They are reliant on their own observations and experience and the patient’s subjective response to the therapy. In addition, no device or system that provides therapists with quantitative evaluation metrics for physiological and musculo-kinetic monitoring and analysis in underwater activities that would allow them to adjust their instructions in real-time to ensure that exercises are being performed correctly with maximum efficiency and impact.

In the absence of precise measurement instruments, quantifying the efficacy of rehabilitation exercises conducted in aquatic environments becomes a challenging endeavor. Therefore, the effectiveness of aquatic rehabilitation has been mostly examined and validated by the comparisons between pre- and post-treatments outside the water; it led to a lack of data for meaningful individual follow-up during patients’ aquatic rehabilitation. These methods are subjective and less include objective data that can provide quantifiable information about muscle activity, movement patterns, and overall performance. This hinders the ability of physiotherapists and exercise professionals to accurately evaluate and tailor aquatic rehabilitation programs for individual needs.

As a result, there is a distinct need for continuous data collection and storage that provides quantitative insights into patients’ movements in the water in order to improve the rehabilitation process. This not only has benefits for patient care, but also for scientific research, data analysis, and further field advancements (Marta et al., 2020). In the other words, a new bioinstrumentation system that encompasses real-time physiological and motor monitoring and analysis during aquatic therapy will lead to a new scientific field that will offer patients personalized rehabilitation treatment to enhance the quality of their performance in therapy. By developing a waterproofed wearable device capable of collecting surface electromyography (sEMG) and inertial measurement unit (IMU) data, this study aims to provide objective and quantitative measurements of muscle activity and movement by designing a system that combines quantitative analysis of movement using sEMG and IMU data-based features during aquatic rehabilitation. These measurements can then be used to analyze the effectiveness of different exercises, identify areas for improvement, and inform the development of personalized aquatic rehabilitation programs.

## 2 Methods and materials

Healthy ten male and five female subjects ([mean ± SD]: age, 25.7 ± 3.1 *years*; height, 167.6 ± 9.6 *cm*; body mass, 68.3±7.6 *kg*) from the University of Massachusetts (UMass) Amherst were recruited for this research. To ensure the validity and dependability of the study’s results, individuals with pre-existing health conditions that could hinder their functional capacities were meticulously screened out of the participant pool. Enrollment in the study was accompanied by informed consent documentation and thorough information on the research objectives and methodologies provided to participants in advance. All subjects agreed to participate in this study and were informed about procedures, potential risks, and use of human images/videos during the experiment. They gave their informed written consent to participate in the study. Institutional Review Board (IRB) approval was obtained from UMass Amherst (#22010038) to carry out the experiments.

### 2.1 Device and electrodes

**FIGURE 1:**
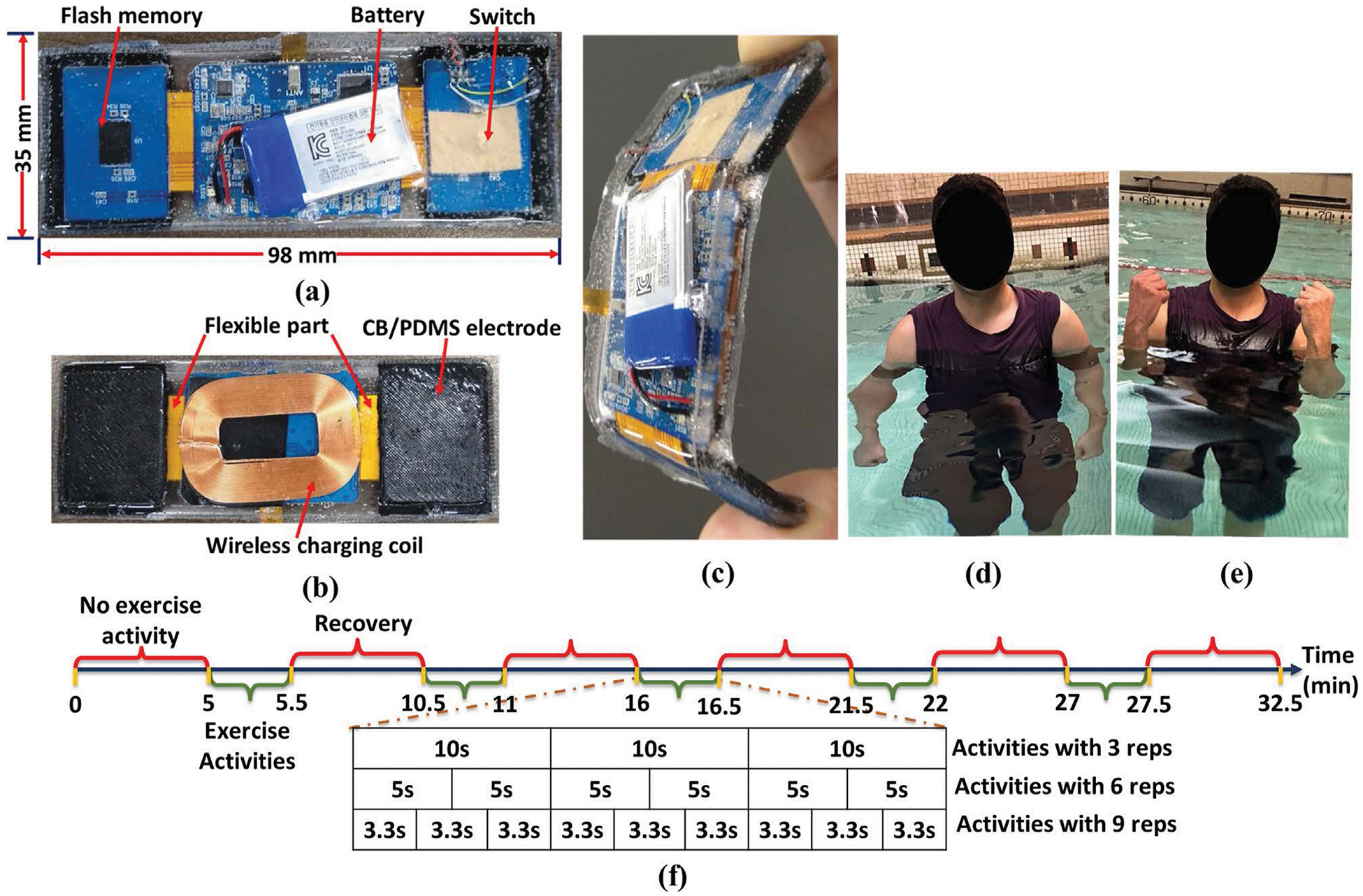
Wearable sEMG and IMU recording device with **(a)** top view, **(b)** CB/PDMS electrodes on two wings connected with flexible part and wireless charging coil (bottom view), **(c)** flexibility of the device (side view), **(d)** extension of the activity- Bicep Curls (BC), **(e)** flexion of the activity- BC, **(f)** timeline of the steps during the BC and TK.

The device consists of two sensing units operating in parallel—the IMU, sEMG sensor unit, including flash memory unit. The device is waterproof and powered up by a 135mAh 3.7V Li-Polymer battery, providing continuous operation for up to 8 h, which is rechargeable wirelessly as it is shown in Figure 1a. The carbon black/polydimethylsiloxane (CB/PDMS) electrodes, as illustrated in Figure 1b and documented in references (Noh et al., 2016; 2018; Posada-Quintero et al., 2018; Amin et al., 2023), are seamlessly linked across both wings *via* a flexible segment. This study embraces CB/PDMS electrodes instead of traditional Ag/AgCl electrodes due to their reliability, waterproof nature, and resistance to motion, which are essential for sEMG signal acquisition in aquatic settings. Traditional Ag/AgCl electrodes, although commonly utilized in dry environments, experience issues such as hydrogel washout, water infiltration, and heightened impedance variability when immersed. CB/PDMS electrodes present a hydrophobic and durable option, demonstrating stable impedance across various water conditions, such as fresh, chlorinated, and saltwater environments (Noh et al., 2018). Prior research indicates that these electrodes are capable of obtaining high-fidelity bio-signals without the necessity of adhesive waterproofing layers, commonly required for Ag/AgCl electrodes (Noh et al., 2016). The superior motion artifact resistance of CB/PDMS electrodes is a significant advantage, particularly for dynamic rehabilitation exercises (Posada-Quintero et al., 2018). In contrast to Ag/AgCl electrodes, which are susceptible to displacement and signal distortion during movement, CB/PDMS electrodes ensure consistent signal acquisition, even during extended immersion and active movement. Our previous research confirmed that CB/PDMS electrodes sustained high-fidelity ECG signals for more than 6 h of continuous underwater application without any material degradation (Noh et al., 2016). Considering these benefits, CB/PDMS electrodes were chosen for this study to facilitate reliable, long-term sEMG monitoring in aquatic settings. Their capacity to preserve signal integrity, resist water infiltration, and reduce motion artifacts renders them optimal for evaluating muscle activity in rehabilitation contexts.

The meticulously designed rigid-flexible structure enhances adaptability, ensuring optimal conformity to the body shape while mitigating the risk of water penetration between skin and electrode and potential data loss. The side view in Figure 1c emphasizes the device’s flexibility, showcasing its ergonomic design for optimal user comfort and functionality. The accelerometric and gyroscopic data from the IMU sensor and 1-channel sEMG data were stored in flash memory with capacity of 128 *Mbit* to utilize for further signal processing and statistical analysis.

### 2.2 Experimental procedure

Dexterous upper-limb impairments are a substantial cause of disability in the aftermath of an acquired brain injury or stroke, affecting approximately half of the patients in this clinical population (Roby-Brami et al., 2021). The restoration of upper-limb function following stroke has been the subject of numerous studies from both fundamental and clinical perspectives. In this context, our research concentrates on the Bicep Brachii (BB) and Tricep Brachii (TB) muscles, which are essential for the control of upper extremity neuromuscular and equilibrium activities. The device was fastened with elastic belt worn around the subject’s bicep and the sensor node was positioned on the Bicep Brachii (BB) and Tricep Brachii (TB) of the subject. Two experiment protocols- Bicep Curls (BC) and Tricep Kickback (TK) were performed on-land and aquatic environments to test the feasibility of the developed device for different numbers of repetitions (reps) in different directions. At the start of protocol 1, subjects were instructed to stand straight with their elbows fully extended and their arms parallel to their bodies as it is illustrated in Figure 1d. Then they were asked to lift the forearm (flexion) with the palm in the upward direction till the full flexion as it is demonstrated in Figure 1e and slowly lowered the forearm (extension) back to the straight position and parallel with the body. On the other hand, in protocol 2, subjects were asked to follow the same exercise, but the device was placed on Tricep. Each activity had 3, 6, and 9 reps in 5 sets, respectively. As it is depicted in Figure 1f, each experiment began with a 5-min resting stage to obtain the baseline of the collected sEMG and IMU data, then transitioned to a contraction stage, which consisted of isotonic contraction for 30 s. We followed the same time sequence for the data recording on both protocols 1 and 2. Before each test, subjects were asked to practice the requested activities until they were familiar with them. All participants accomplished the protocols successfully, and completed activities without any physical and mental issues in both categories.

### 2.3 Feature extraction

In the analysis of the sEMG signal, features are predominantly categorized into three main representations: time domain, frequency domain, and time-frequency/time-scale representation (Du and Vuskovic, 2004; Oskoei and Hu, 2007; Zecca et al., 2002; Oskoei and Hu, 2008). In this study, we conducted only first two feature groups, which are defined in time domain and frequency domain analysis for sEMG signal in this study, have been considered because since the features in the last group, time-frequency/time-scale features, representation cannot be directly used by themselves for the musculoskeletal interventions in aquatic therapy and rehabilitation (Englehart et al., 2001; Phinyomark et al., 2009). Features extracted from time-frequency/time-scale methods representation should be reduced to their high dimensions before sending them to a classifier. Additionally, mathematical functions which were defined in time domain and frequency domain have been usually used as dimensionality reduction methods for time-frequency/time-scale domain features (Boostani and Moradi, 2003). Hence, study of feature extraction properties of time domain and frequency domain has recently become an important issue in the sEMG signal classification. There are twelve features that were used in this evaluation study presented in Table1 (Du and Vuskovic, 2004; Oskoei and Hu, 2007; Zecca et al., 2002; Oskoei and Hu, 2008). A baseline is established for healthy individuals by determining the normal range of feature values through our experiments in two distinct environments, following the given protocols. 6 sEMG features in the time domain and 3 ones in the frequency domain were computed in this study, and their mathematical definitions and the related works are listed in Table1 (Phinyomark et al., 2009; Rafiee et al., 2011).

**FIGURE 2:**
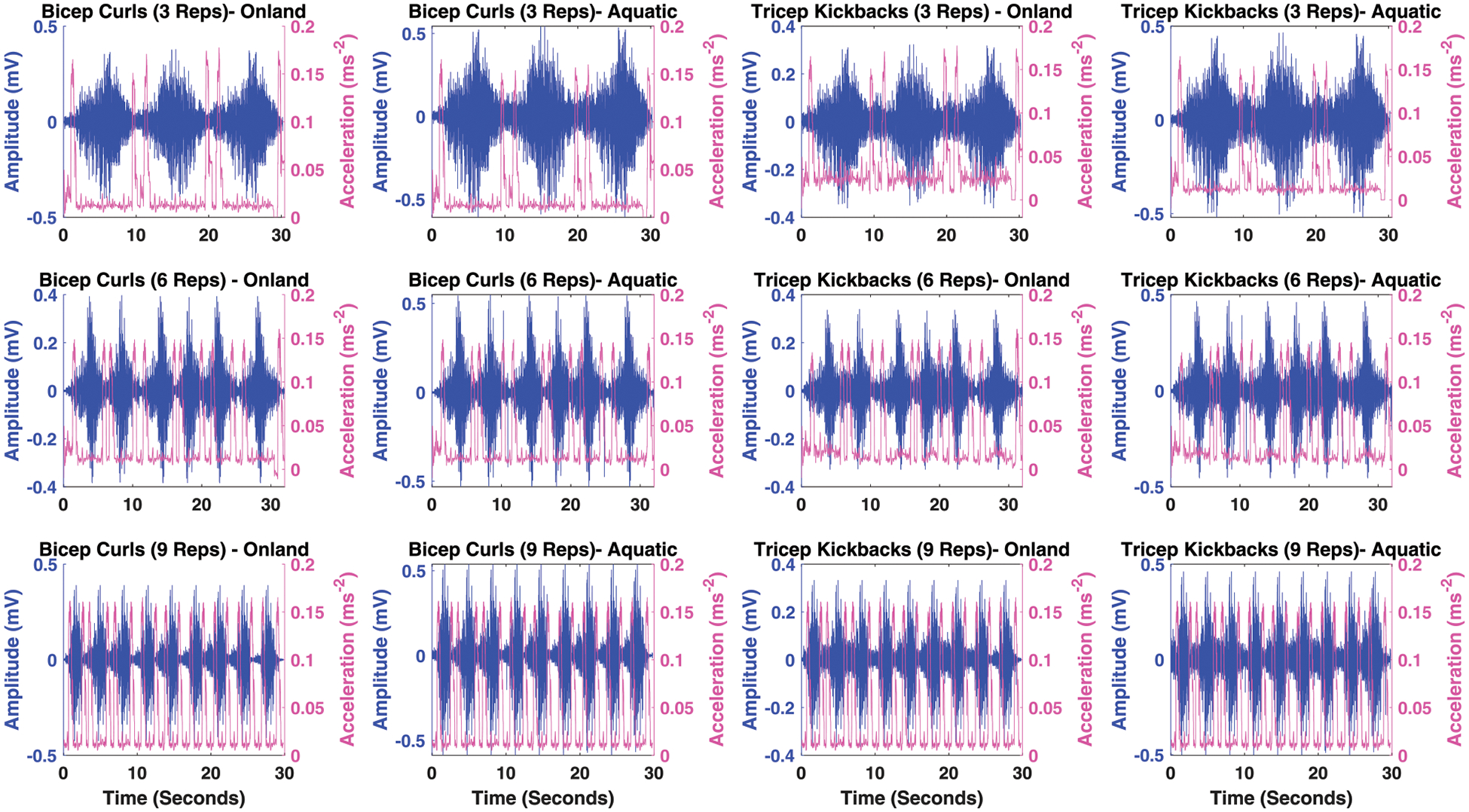
Illustration of sEMG for muscle activity and limb movement acceleration across BC and TK activities for 3, 6 and 9 reps in on-land and aquatic environments.

As it is illustrated in Figure 2, the increase in acceleration is utilized as an indication of the initiation of arm extension and flexion movements. This specific feature is employed to determine the start and end of limb movement. Consequently, the device is programmed to commence the collection of sEMG data upon detecting an increase in acceleration, indicative of the onset of activity. It then ceases data collection at the next instance of acceleration increase, marking the completion of the activity. This method ensures efficient and targeted data acquisition, focusing on the periods of active muscle engagement. The sEMG signals underwent normalization using the Maximal Voluntary Contraction (MVC) as a reference value, obtained from the same muscle during MVC. Following normalization, the sEMG signals underwent further processing through rectification and smoothing via the calculation of the root mean square (RMS) of the signal. Descriptive statistics, encompassing mean and standard deviation, were computed for all the defined features in Table 1 across both protocols and both environments (land and aquatic).

In order to evaluate the differences in sEMG characteristics between distinct testing circumstances (on-land and aquatic environments) for each protocol, an independent (two-sample) *t* – *test* with repeated measures was utilized. This statistical technique is employed to ascertain whether there are any statistically significant disparities between the means of on-land and aquatic groups which are corresponding to hypothesis, *Null Hypothesis*, (*H*_0_): the group means are significantly different and *Alternative Hypothesis* (*H*_*A*_): the group means are not different. The purpose of our study was to use a *t* – *test* to compare the means of the sEMG features in different surroundings. The process entailed determining the average of each sEMG characteristic for every testing circumstance, thereafter calculating the fluctuation within each group (within-subject fluctuation) and the fluctuation between the groups (between-subject fluctuation). The F-test was subsequently employed to ascertain whether the variability among groups was considerably larger than the variability within groups, so showing a noteworthy disparity between the means of distinct situations. A *p* – *value* below 0.05 in the *t* – *test* shows the evidence to reject the null hypothesis corresponding to the testing conditions.

The reproducibility of sEMG characteristics between testing circumstances was assessed by calculating the intra-class correlation coefficient (*ICC*) for each protocol. The ICC quantifies the degree of dependability or consistency between measurements conducted by several observers when assessing the same variable. Within this particular framework, it measures the degree to which the same values of sEMG characteristics can be acquired when subjected to varying testing settings, hence determining the reproducibility of these features. ICC values closer to 1 suggest a higher dependability and reproducibility in the measurements, which can be considered excellent in our study. Furthermore, the coefficient of variance (*CV*%) was used to provide information on the variability within subjects under different testing conditions. The *CV*% is determined by dividing the standard deviation by the mean and then expressing it as a percentage. The *CV*% values are utilized to quantify the precision of the sEMG characteristics. Lower *CV*% values indicate greater precision and consistency in the readings.

The utilization of *t* – *test*, *ICC*, *CV*% in this comprehensive method offers a strong framework for comprehending and interpreting the results of the quantitative analysis of the sEMG signal conducted using *MATLAB R*2024*b*. The utilization of these statistical techniques guarantees a comprehensive assessment of the data’s dependability, accuracy, and importance, providing a strong basis for the study’s findings.

## 3 Results

The IEMG is employed to assess the pre-activation index of a muscle which is defined by the area under the rectified curve (Babu et al., 2022; Cardoso et al., 2017). The Mean absolute value (MAV) is calculated by taking the average of the absolute values of the sEMG signal within a designated time frame. Remarkably, a significant surge in this feature is observed at the onset and persists at elevated levels throughout the contraction period (Zecca et al., 2002; Abbaspour et al., 2020). The integration of the squared sEMG signal values yields the SSI which represents the energy characteristics of sEMG signals (Jie et al., 2021). RMS is intricately linked to both muscle contraction force and the state of muscle fatigue, serving as a valuable metric in assessing and characterizing these physiological aspects. Moreover, MDF serves as a frequency domain indicator of muscle fatigue during isotonic contraction, with a documented correlation indicating that a decrease in MDF alongside an increase in sEMG signal amplitude serves as a reliable fatigue indicator (Jie et al., 2021; Cao et al., 2017). The Average amplitude change (AAC) is to observe the average variation in the signal amplitude during the contraction period (Aviles et al., 2023). The VAR serves as a quantitative measure for both the concentration and dispersion of signal data values, functioning as an index of signal energy (Jie et al., 2021). Mean Power Frequency (MNF) and Median Frequency (MDF), reflecting average and median power spectrum frequencies, respectively, are sensitive indicators of shifts in firing rates and recruitment patterns associated with fatigue. PKF highlights dominant frequency components, while MNP offers a comprehensive view of muscle activity dynamics during varying fatigue states. These features, extracted from the Power Spectral Density (PSD) of sEMG signals, collectively contribute to understanding the subtle interplay of neuromuscular factors in fatigue (Corvini and Conforto, 2022).

**FIGURE 3:**
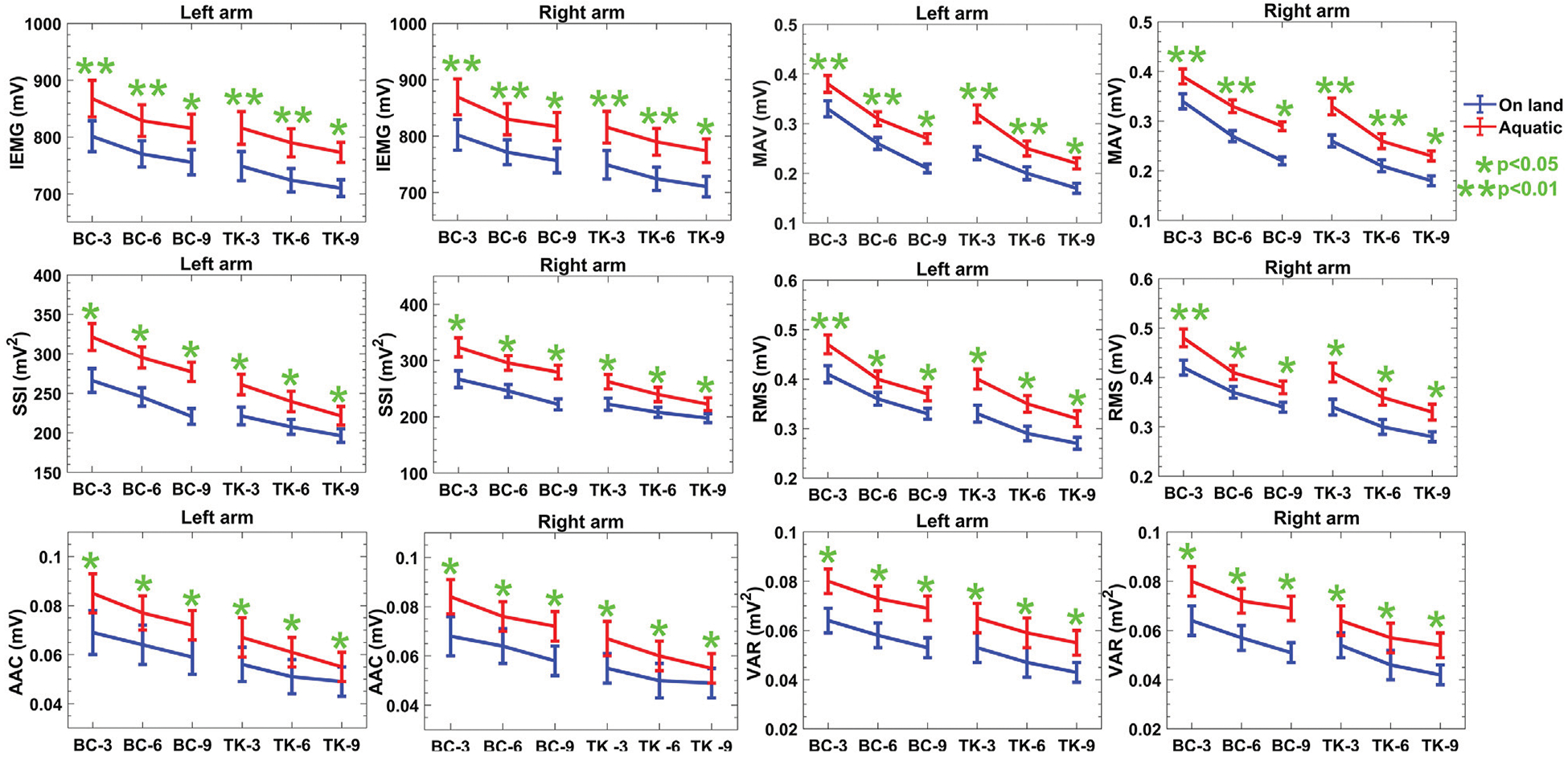
Descriptive statistics (mean ± SD) of both protocols of sEMG time domain features over the reps of the activities in both environments. *p – values* indicate the statistical significance of the comparison between the on-land and aquatic data. BC-3, BC-6, BC-9 denote bicep curls for 3, 6, and 9 reps, while TK-3, TK-6, TK-9 represent triceps kickbacks for the same series of reps, respectively.

The time-domain properties listed in Figure 3 and Table 2 demonstrate a decrease in mean values as the number of repetitions during on-land activities increases, without indicating any notable fatigue as no additional load is applied. Furthermore, it is expected that there would be no substantial variation in the data across the two arms in both protocols for both conditions. Nevertheless, the anticipation could vary for activities conducted in aquatic condition, since the presence of resistance and drag force may result in elevated readings, suggesting heightened muscular exertion. Both on-land and aquatic conditions are expected to show an increasing trend in the frequency domain, indicating an increase in motor unit recruitment and potential adaptations to hydrodynamic pressures. The IMU-based characteristics exhibit an upward trend in acceleration and gyroscope readings, with no notable distinction between on-land and aquatic environments. Ultimately, using this approach, reproducibility metrics will be generated to assess the feasibility of the designed device for acquiring sEMG data in aquatic conditions.

The outcomes of an quantitative comparison between on-land and aquatic environments are detailed in this section. Data were initially collected for both environments. The outcomes of TK and BC executed with both limbs under each environment are subsequently described in detail. Ultimately, a comprehensive comparison is presented, which includes every evaluated aspect of the two environments.

These extracted features provide a concise summary of the descriptive statistics, *t* – *test* results, *ICC*, and *CV*%. It is worth mentioning that there were no substantial variations in Maximum Voluntary Contraction (MVC) scores across the environments for any of the muscles that were monitored. The *CV*% scores for all muscles fell within the range of 2.7% – 6.4%, and ICC values ranged from *r* = 0.93 – 0.98; these results demonstrate the consistency and reproducibility of the measurements across various environmental environments. Measurement of sEMG reproducibility, which means how much it is following the trend of on-land in aquatic. In all cases, the test statistic has an F-distribution with k −1 numerator degrees of freedom, and N -k denominator degrees of freedom.

### 3.1 Comparison of features in the time domain

It is apparent from the time domain analysis that all features in the time domain are decreasing over repetitions of the activities. In the case of both Protocol 1 and 2, amplitude of the features gets intensified in aquatic environment at least 6% compared to on-land one. Table 2 shows the quantitative values of the features in two protocols. Based on the obtained results, the correlation and reproducibility testing are done to verify the acquired data in aquatic environments compared to on-land one. According to Figure 3, evidence exists to reject the null hypothesis about the time domain features found in the two environments, as indicated by the *p*-value, with activities conducted under a no-load condition. However, the presence of female respondents leads to a decrease of around 2 – 5% in the mean values of the features, as well as an increase in the standard deviation (SD) of the features when compared to data solely from male subjects.

### 3.2 Comparison of features in the frequency domain

**FIGURE 4:**
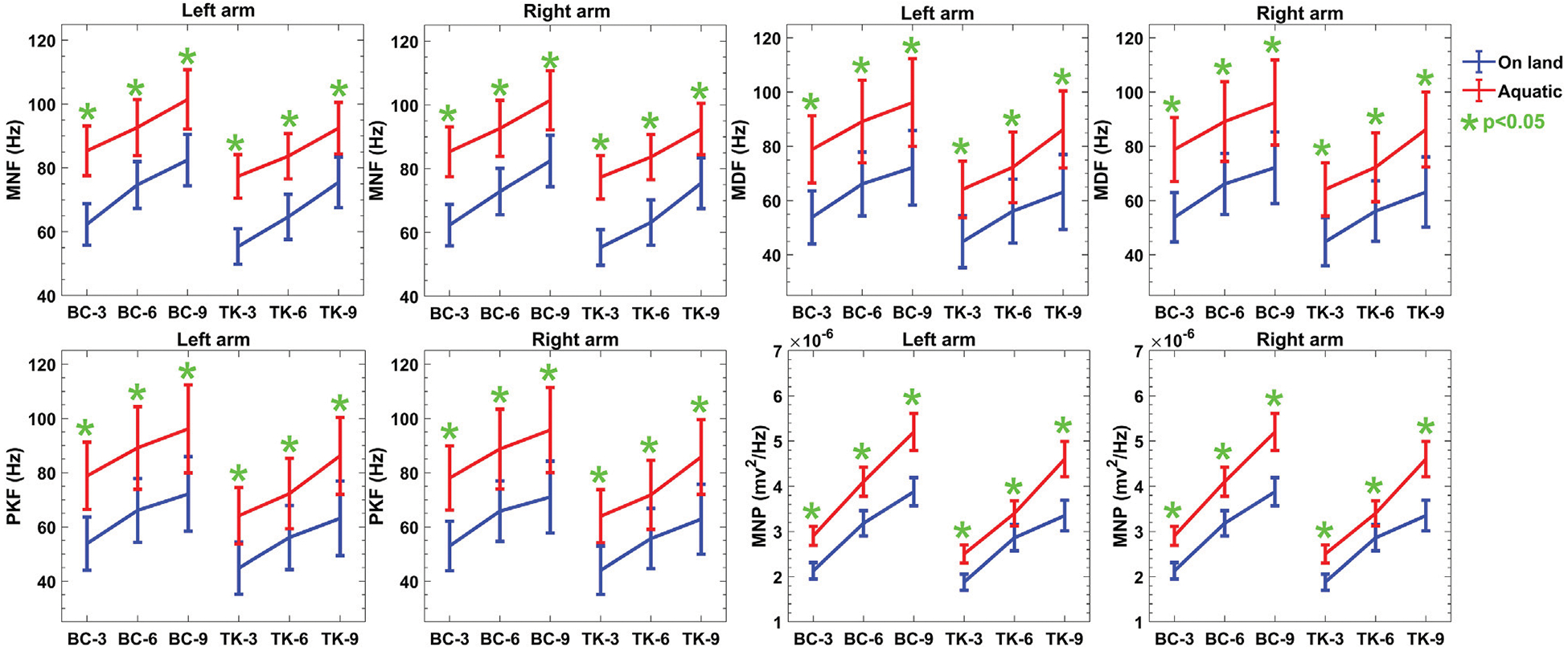
Descriptive statistics (mean ± SD) of both protocols of sEMG frequency domain features over the reps of the activities in both environments. p-values indicates the evidence of rejection of the null hypothesis corresponding to the comparison between the on-land and aquatic data mean value.

In the frequency domain features, an increase in movement speed is likely to result in a shift toward higher frequencies in the sEMG signal, reflected in the median frequency. Moreover, the increased drag force with increased reps in aquatic environments results in a higher frequency of motor unit firing, further contributing to the observed increase in median frequency which is presented in Figure 4. The MNF and PKF show that protocol 2 is more than 21% larger than protocol 1 in both environments. However, in MNF, protocol 2 shows a lower value than protocol 1.

### 3.3 IMU feature for kinematics

**FIGURE 5:**
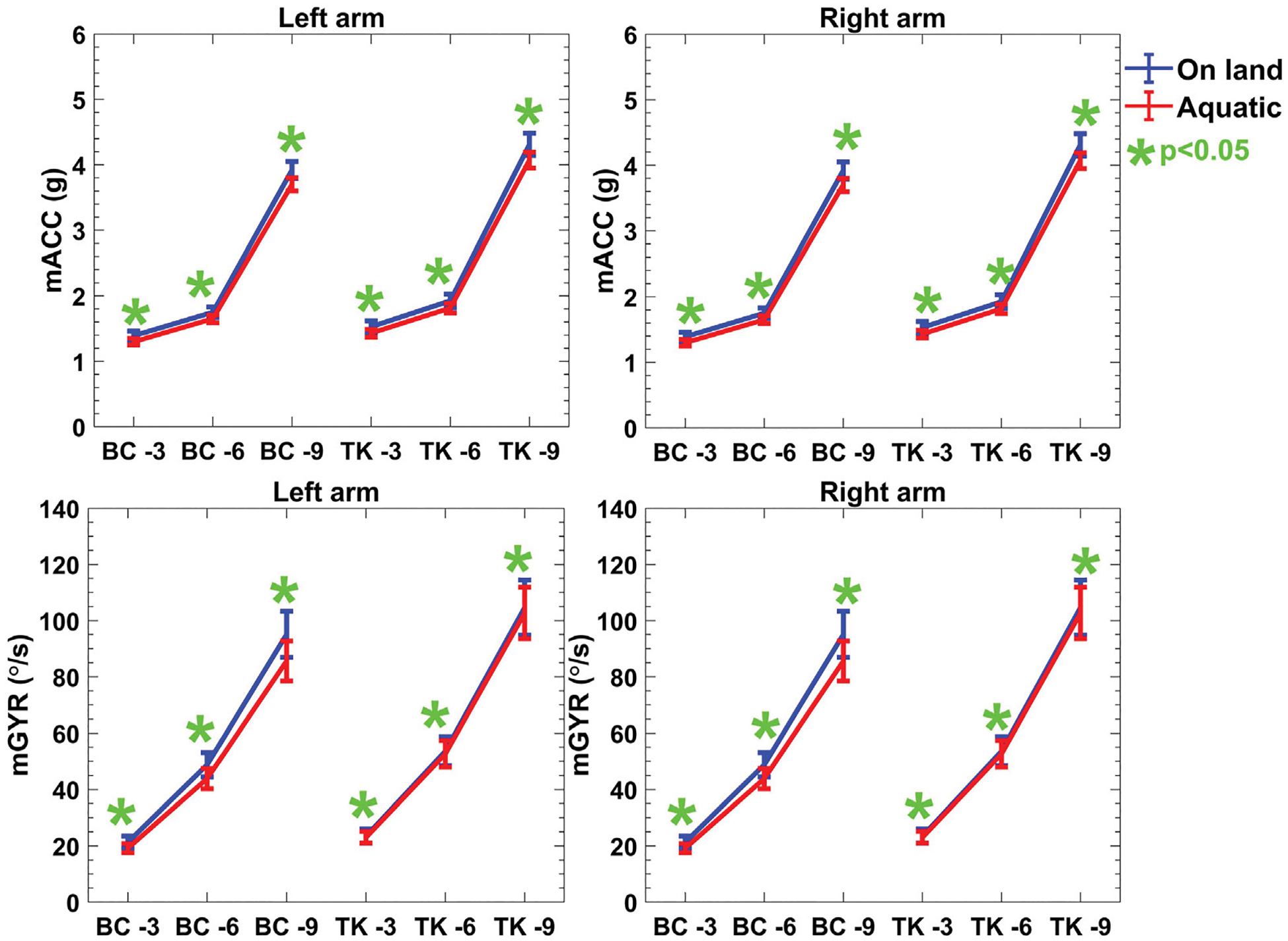
Descriptive statistics (mean ± SD) of both protocols of kinematics features over the reps of the activities in both environments. p-values indicates the evidence of rejection of the null hypothesis corresponding to the comparison between the on-land and aquatic data mean value.

Accelerometer data were bandpass filtered to remove both high frequency noise and unwanted gravitational acceleration. Here, in same environments, there is no significant difference in acceleration of arm movements between two protocols as the subjects were guided to move in same way. However, the magnitude of mACC is reduced more than 10% in both protocols which is observed in Figure 5. Analysis of the IMU-derived mACC showed that movement velocity increased in line with an increase in repetition frequency within the same time window. This indicates that participants increased their repetition speed over time, resulting in elevated mACC values. Simultaneously, IEMG exhibited a decline, signifying diminished muscle activation during that timeframe as a result of reduced contraction intensity. This trend indicates that reduced resistance from lower drag forces correlates with decreased muscle effort, consistent with prior research showing that increased resistance generally results in heightened muscle activation.

### 3.4 Reproducibility and reliability of the features

**FIGURE 6:**
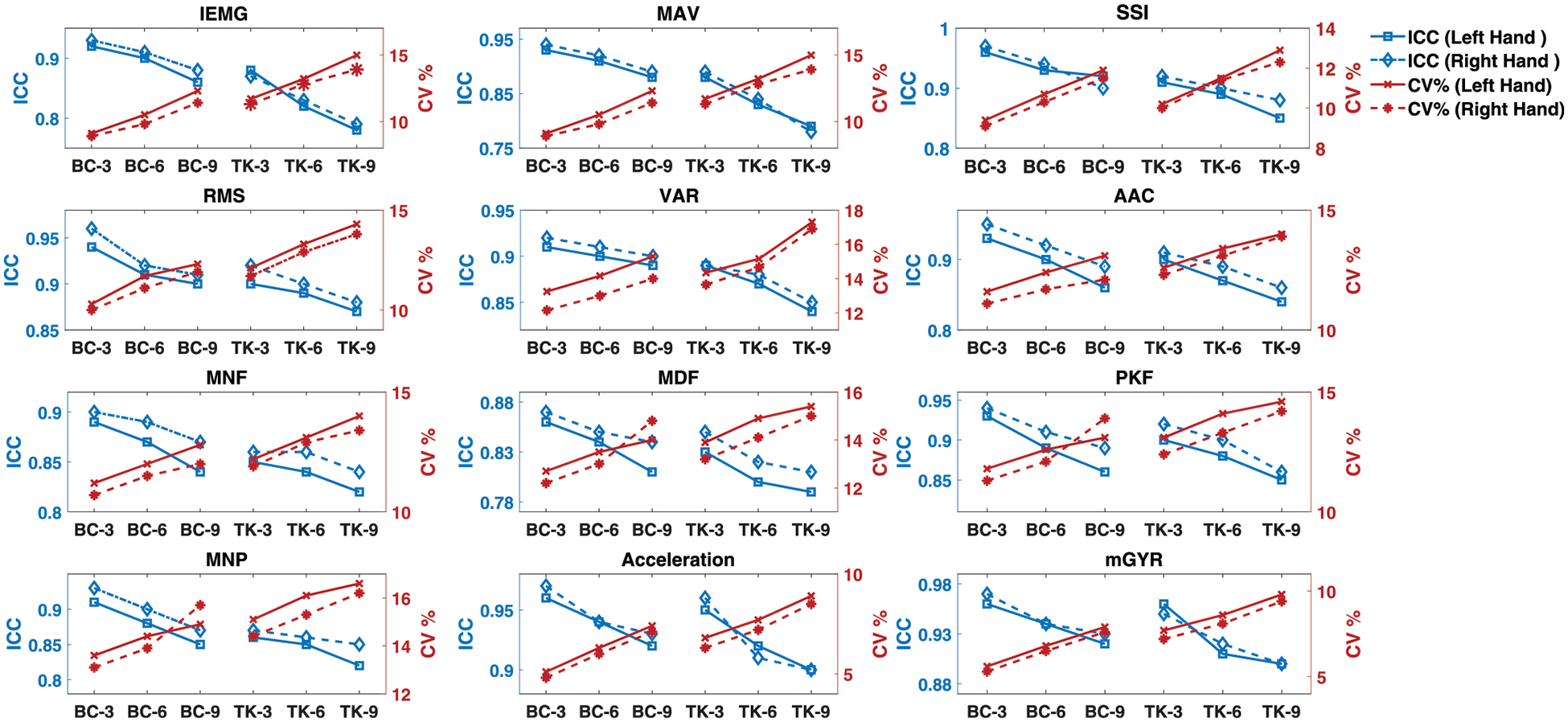
Descriptive statistics (mean ± SD) of both protocols of kinematics features over the reps of the activities in both environments. *p*-values indicates the evidence of rejection of the null hypothesis corresponding to the comparison between the on-land and aquatic data mean value.

In case of time domain features, ICC and CV% ranged between *r* = 0.93 – 0.98 and 2.7– 6.4%, respectively, between environments for the two protocols which is presented in Figure 6. On the other hand, the AAC shows the overall amplitude change which is 11.21% bigger in the case of protocol 1 than the protocol 2. Finally, though the amplitude of the sEMG is higher in aquatic environment, the time domain features *p*-values < 5% is illustrating the evidence of rejection of the null hypothesis corresponding to the comparison between the on-land and aquatic data mean value. Meanwhile, all the frequency spectrum features show the agreement and correlation between on-land and aquatic environments based on *ICC* and intra-subject *CV*% which were reported to evaluate sEMG reproducibility and precision, respectively, and shown in Figure 6. Reproducibility and precision of the sEMG recordings for each muscle in the study were obtained high (ICC = 0.92–0.96, CV% = 5.4–13.8%). These findings are in agreement with the activities performed similarly with others who have performed similar reproducibility trials on-land and in aquatic environments (Silvers and Dolny, 2011; Pöyhönen et al., 1999). Here, the frequency domain features were observed high on land-to-aquatic (ICC = 0.95–0.99, CV% = 3.5–11%) and reproducibility (ICC = 0.85–0.98, CV% = 11–18%) for both protocols.

## 4 Discussion

In technology-assisted rehabilitation, many extant systems integrate various forms of visual and potentially multimodal feedback (Marta et al., 2020; Komar et al., 2012). This feedback-based system, which uses external signals, typically visual or auditory, to facilitate the process of motor learning, is a valuable technique in this domain. This entails the transient generation of information about a patient’s performance, intending to improve their motor function control. It serves a dual purpose: firstly, it facilitates patients in acquiring more significant control over their motor functions, and secondly, it provides quantitative assessment parameters for therapists. As a result, this system improves precision during functional tasks, increases patient adherence to rehabilitation programs, and decreases the need for continuous monitoring by healthcare professionals. This innovation can potentially enhance the overall effectiveness and accessibility of rehabilitation efforts (Kaneda et al., 2013).

### 4.1 Comparison of sEMG-IMU features on-land and aquatic environments

In our investigation of muscle activation patterns during BC and TK, time-domain features, presented in Figure 3, revealed a consistent downward trend in mean values over increasing repetitions, both on-land and aquatic environments. This reduction could be attributed to fatigue accumulates in the involved muscle groups due to repeated BC and TK activities. The progressive decrease in time-domain features suggests that muscles may exhibit reduced activation levels over successive repetitions due to fatigue. The statistical significance uncovered by *t* – *test* implies that aquatic conditions induce unique neuromuscular responses, evident in the increased feature values. For instance, the aquatic setting consistently exhibited higher values suggesting that the added resistance and drag forces in water contribute to elevated muscle activation levels. The *p*-values for these comparisons were less than 1%, reinforcing that no significant differences observed.

Moreover, in the frequency domain depicted in Figure 4 of sEMG exhibited an upward trend over increasing repetitions, both on-land and aquatic environments. This trend suggests a modulation in muscle recruitment patterns, likely influenced by heightened resistance and drag forces in the aquatic environment (Yokoyama et al., 2021). The observed differences between on-land and aquatic feature values underscore the sensitivity of these metrics to environmental context. For example, the increased mean power of sEMG in water may reflect the need for additional muscle recruitment to overcome the resistance, leading to higher frequency components in the signal. These insights highlight the relevance of fatigue and drag forces in shaping the frequency domain response during aquatic activities. The *p*-values for these frequency domain comparisons were consistently less than 5%, signifying that no statistical differences were observed between on-land and aquatic data feature set.

Examining IMU-based features presented in Figure 5, including mACC and mGYR values, further revealed an upward trend with increasing repetitions in both on-land and aquatic conditions. Here, no significant difference was observed between on-land and aquatic environments. Notably, for both acceleration and mean gyroscope values, the on-land measurements consistently exceeded their aquatic counterparts, reflecting the additional load for moving the limbs presented by the aquatic medium. The *p*-values for these IMU-based feature comparisons were consistently less than 5% for Reps 3, 6 and 9, highlighting the statistical significance of the evidence of the rejecting null hypothesis (*H*_0_).

In our assessment of the reproducibility, correlation, and agreement between on-land and aquatic features, we observed encouraging outcomes indicating strong correlation, reproducibility, and agreement. While there is a general declining trend in *ICC* values, coupled with an increasing trend in *CV*%, it is crucial to note that our features, particularly *ICC* values, demonstrate robust correlations. For instance, the *ICC* values for IEMG show consistently positive correlations even with repetitions, signifying good measurement reliability over time. The concurrent rise in *CV*% across features, though indicative of growing variability, should be contextualized within the overall positive framework of our findings. This underscores the importance of monitoring and accounting for factors such as fatigue, reinforcing the need for meticulous data interpretation in research and clinical applications. As we carefully evaluate the feasibility of aquatic data collection and ensure the accuracy of measurements compared to on-land scenarios, these subtle trends affirm the establishment of reliable protocols for assessing movement accuracy in aquatic rehabilitation settings. These insights not only provide therapists with valuable information for guiding patients through their rehabilitation journeys but also support the broader application of quantitative measurements in aquatic contexts.

The utilization of aquatic sEMG recordings has produced persuasive results demonstrating a significant increase in amplitudes of signals and sEMG/force ratios during isotonic muscle contractions in comparison to on-land measurements. There have been numerous hypotheses put forth in an attempt to explain the documented increases in muscle activity and force output that occur during water immersion. However, there is one theory posits that these alterations could be ascribed to the impaired operation of specific reflex mechanisms or to a compensatory mechanism within the muscles that impact their ability to generate force (Silvers and Dolny, 2011; Pöyhönen and Avela, 2002). Furthermore, it has been determined that water infiltration onto electrode attachments or wires may also contribute to diminished sEMG amplitudes. Previous studies have indicated that although attempts have been made to insulate electrodes, the electrical output of human musculature may inherently decrease when exposed to water.

Moreover, submerged sEMG recordings may be contaminated by the effect of water on the skin’s surface, which alters the resistance of the electrical surface Coulange et al. (2008). Significantly, the impact of buoyancy-induced weightlessness on the neuromuscular system, specifically on proprioceptive systems and muscle spindles, could potentially have a critical influence on the amplitudes of sEMGs during voluntary contractions that are either maximal or submaximal. Therefore, in analyzing the results of the current investigation, it is critical to account for the intricate interaction of these diverse elements within the sub-aqueous environment.

We found that the amplitudes of sEMG recordings (RMS, MAV and ARV) from the BB during contractions were increased to 18.7 ± 3.1% of similar muscle contractions recorded in aquatic environments compared to on-land. In the evaluation of sEMG reproducibility and precision across three MVC tests for bicep and tricep muscles in Figure 6, ICC and CV% were employed, following methodologies from previous research (Norcross et al., 2010; Rainoldi et al., 2001). This study established that our system can consistently and effectively acquire data in aquatic environments, maintaining the reproducibility observed in on-land conditions. This is substantiated by Figure 6, which displays significant correlations in extracted features (*p* < 0.05), underscoring the system’s robust and reliable performance across different rehabilitation environments.

### 4.2 Real-world implications of real-time tracking motion and muscle activation in aquatic rehabilitation

One practical advantage of the proposed system in this study is its standalone nature and convenient location, making it suitable for load-bearing activities without hindering task performance, aligning with principles of ergonomic design (Webber and Rojas, 2021; Shukla et al., 2020). However, its limitation lies in its sole focus on forearm motion, preventing the measurement of intersegment or full-body movements. Notably, no single wearable sensor modality can comprehensively capture all aspects of motor behavior. IMU sensors are sensitive to motion but lack deterministic connections to force generation, while sEMG measures muscle activation but is not directly linked to motion. As technology evolves and more sensors capable of quantifying various aspects of motor behavior become available, the understanding of the relationships between sensor data and function is likely to improve. By combining machine learning classification methods and multimodal performance data, the development of more effective algorithms for task discrimination and better measures for assessing activities of daily living (ADL) and aquatic activities performance is a possibility. It’s important to note that the classifiers were not tested on additional gestures outside the presented protocols and repetitions, and future work should explore their robustness through evaluation on a broader set of gestures to provide additional performance metrics.

Our results are in close alignment with the current clinical practices for upper limb rehabilitation, particularly in the context of stroke rehabilitation. By offering precise and real-time feedback on muscle activation patterns, the integration of our device can improve the efficacy of therapeutic exercises, thereby supporting established rehabilitation protocols. Our device can be seamlessly integrated into existing clinical operations, thereby fostering personalized and data-driven rehabilitation programs, by adhering to these protocols.

### 4.3 Activity category prediction through movement and muscle activation

Though mACC does not differ across task significantly, the sEMG features are differing across the protocols and environments. These results are consistent with the mechanisms by which the tasks were originally separated. An accelerometer filtering and thresholding approach was used in (Uswatte and Hobbs Qadri, 2009; Totty and Wade, 2017). Because acceleration was used, we expected it to be useful in distinguishing between protocol activity classes. Here the sEMG distinguishing features are higher for Protocol 1 and 2 in aquatic environment; this is likely due to the nature of drag force of the water, which exhibits low acceleration. The clusters are constructed by the combination of quantitative outcome of sEMG, mACC and mGYR. Eventually, though the mACC shows similar characteristics over the protocols and environments, sEMG can classify the protocols activities. The more vigorous motion of Protocol 1- Reps 9 represented the highest values of mACC and mGYR which was anticipated based on the increased intensity of the chosen tasks and it is shown in Figure 5.

It is critical to improve modified and more precise rehabilitation by integrating a classification technique with adaptive learning and real-time analysis. Our system is equipped with the capability of real-time monitoring *via* a microcontroller’s quantitative computation capacity and concurrent data storage in flash memory for future use. Moreover, in order to encourage additional classification research, the clustering of the data utilizing characteristics from sEMG-IMU datasets is presented. Hence, by incorporating adaptive training models into the design of this proposed system, aquatic rehabilitation can be enhanced further. As part of this classification endeavor, we presented the clustering of the healthy persons dataset’s features.

## 5 Challenges and opportunities

Our research makes a substantial contribution to the comprehension of the use of our wearable device for aquatic rehabilitation, as it demonstrates its capacity to collect data in aquatic environments with reliability. The research establishes a strong foundation for future investigation. The findings provide valuable insights and underscore the potential for a more extensive application. The external validity and generalizability of our findings will be improved in future research by diversifying the sample size in terms of gender and age within the healthy population. Moreover, the restricted scope to a homogeneous cohort of healthy participants might impede the direct generalizability of our results to clinical populations, particularly individuals afflicted with neurological or orthopedic disorders. The assessment of the device’s efficacy solely in relation to BC and TK protocols constitutes a fundamental stage. Nevertheless, it is acknowledged that further research is warranted to investigate a wider range of exercises frequently utilized in aquatic rehabilitation. Furthermore, our research provides substantial insights into the environmental impact and short- and long-term viability of the wearable device. The longevity, user-friendliness, and comfort of our device have been evaluated in accordance with the established protocol. The practical outcomes of the device in aquatic rehabilitation situations are more comprehensively understood as a result of these tests.

With a focus on combining technology and human expertise, our research’s future directions offer promising avenues for expansion and improvement. In order to incorporate the wearable system into clinical practice, we have to go through following steps. Initially, carry out preliminary research in partnership with rehabilitation clinics to evaluate the practicality and efficacy of the wearable device in authentic environments. We shall adhere to conventional physiotherapy techniques for people with mobility issues. Using these established protocols, we will collect feedback from both therapists and patients. We will then score and categorize our dataset with the assistance of physiotherapists. Additionally, establish training initiatives for therapists to guarantee their competence in using the wearable gadget and analyzing the data. This training will encompass the technical intricacies of the device, along with the most effective methods for incorporating it into therapeutic sessions. Furthermore, focus on the task of merging the data obtained from the wearable device with the current clinical information systems. This would enable smooth and uninterrupted interchange of data, hence improving the entire workflow in rehabilitation settings. Lastly, it is important to recognize and overcome any obstacles that may hinder the implementation process. These barriers include guaranteeing the comfort and user-friendliness of the device, effectively maintaining the privacy and security of data, and ensuring that the cost is reasonable enough to encourage wider adoption. By adhering to these procedures, the wearable device can be seamlessly incorporated into clinical practices, offering a potent instrument for augmenting rehabilitation therapy and optimizing patient results. This quantitative analysis even highlights the potential for real-time monitoring, paving the way for continuous and adaptive system assessment (Amin et al., 2023).

Incorporating machine learning techniques can significantly enhance the capabilities of our wearable device, enabling real-time data analysis and personalized feedback for users. Furthermore, we envision the inclusion of therapists in the loop, where their clinical insights and expertise play a pivotal role in refining the algorithms and tailoring recommendations to individual patient needs. By fostering a collaborative approach between technology and human expertise, we aim to develop a classification system that not only automatically recognizes and assesses different aquatic exercises but also incorporates valuable input from rehabilitation professionals. This collaborative model not only ensures the accuracy of exercise classification but also leverages the unique skills of therapists in interpreting patients’ responses. Such innovations align with the evolving landscape of wearable technology, transforming our device into a dynamic tool for tailored interventions that seamlessly integrate the wisdom of healthcare professionals into the digital realm. In our upcoming phase, by employing the Long Short-Term Memory (LSTM) network (Amin et al., 2023), we can gain a deeper comprehension of the musculo-kinetic patterns displayed by patients during aquatic therapy. This will lead to the creation of rehabilitation programs that are not only more effective but also customized to meet individual requirements. Furthermore, the incorporation of machine learning for classification recommendations has the potential to facilitate the development of adaptive training programs, which can cater to individual differences in movement patterns and rehabilitation progress. These developments enhance the ongoing development of our wearable technology and promote a more inclusive, intelligent, and cooperative approach to aquatic therapy.

## 6 Conclusion

Here, we describe a prototype of a wearable device—unique among its kind—capable of quantifying patient movements during aquatic rehabilitation, transferring data to a location other than water to facilitate prospective physiotherapist monitoring, and storing data for subsequent analysis. The research aimed to test and verify the effectiveness of a wearable device in aquatic environments for rehabilitation exercises like BC and TK. The study focused on analyzing sEMG and IMU data to categorize activities and identify the start and end points of exercises based on limb movement acceleration. The findings confirm the key idea that this approach is viable for precise, real-time monitoring in aquatic rehabilitation, enhancing exercise classification and providing valuable insights for therapists in tailoring patient-specific rehabilitation programs. Design-wise, robust real-time feedback utilizing classification techniques, and a machine learning strategy based on quantitative analysis are still areas that require improvement.

## Figures and Tables

**TABLE 1:** List of features extracted with definitions and significance (Babu et al., 2022; Cardoso et al., 2017; Abbaspour et al., 2020; Jie et al., 2021).

	Features	Model	Normal range	Unit	Note
Time domain	Integrated EMG, (IEMG)	$\sum_{i=1}^{N}\left|x_{i}\right|$	700 ≤ *R* ≤ 900	*mV*	comprehensive measure of muscle activity
	Mean absolute value, (MAV)	$\frac{1}{N}\sum_{i=1}^{N}\left|x_{i}\right|$	0.1 ≤ *R* ≤ 0.4	*mV*	strength of muscle contractions
	Simple square integral (SSI)	$\sum_{i=1}^{N}x_{i}$	200 ≤ *R* ≤ 350	*mV* ^2^	energy of the sEMG signal
	Root mean square (RMS)	$\sqrt{\frac{1}{N}\sum_{i=1}^{N}x_{i}^{2}}$	0.1 ≤ *R* ≤ 0.5	*mV*	onset and progression of muscle fatigue
	Average amplitude change (AAC)	$\frac{1}{N}\sum_{i=1}^{N-1}\left|x_{i+1}-x_{i}\right|$	0.01 ≤ *R* ≤ 0.1	*mV*	Variation of muscle activity under different circumstances
	Variance of sEMG (VAR)	$\frac{1}{N-1}\sum_{i=1}^{N}x_{i}^{2}$	0.01 ≤ *R* ≤ 0.1	*mV* ^2^	variability in muscle contractions
Frequency domain	Mean Power Frequency (MNF)	$\frac{\sum_{j=1}^{M}f_{i}P_{j}}{\sum_{j=1}^{M}P_{j}}$	40 ≤ *R* ≤ 100	*Hz*	indicative of muscle fatigue
	Median Frequency (MDF)	$\sum_{j=1}^{MDF}P_{j}=\frac{\sum_{j=1}^{M}P_{j}}{2}=\sum_{j=MDF}^{M}P_{j}$	40 ≤ *R* ≤ 100	*Hz*	Modulation of muscle activity for specific actions
	Peak frequency (PKF)	$max\left(P_{j}\right)$	40 ≤ *R* ≤ 100	*Hz*	dominant frequency
IMU	mACC	$\sqrt{Acc_{x}^{2}+Acc_{x}^{y}+Acc_{x}^{z}}$	1 ≤ *R* ≤ 6	*g*	Limb motion acceleration
	mGYR	$\sqrt{Gyr_{x}^{2}+Gyr_{x}^{y}+Gyr_{x}^{z}}$	0 ≤ *R* ≤ 100	°/*sec*	Limb motion velocity

**TABLE 2:** Quantitative result of features for both protocols in both conditions.

Features	Environment	Protocols	Arms					
			Left			Right		
			3	6	9	3	6	9
IEMG (*mV*)	Land	BC	801.173± 27.31	770.07± 23.16	755.45± 22.09	802.05± 27.19	771.22±22.09	756.30± 21.83
		TK	748.88± 25.62	723.86± 21.62	709.65± 18.78	748.28± 25.33	724.16± 20.69	710.12± 17.98
	Aquatic	BC	867.76± 32.16	828.86± 28.01	815.42± 25.02	869.78± 31.88	830.06± 27.82	816.92± 24.82
		TK	815.89± 28.88	789.73± 24.90	772.82± 21.74	816.38± 28.16	790.09± 23.94	773.82± 21.07
MAV (*mV*)	Land	BC	0.33± 0.016	0.26± 0.012	0.21± 0.009	0.34± 0.015	0.27± 0.011	0.22± 0.008
		TK	0.24± 0.013	0.2 ± 0.013	0.17± 0.01	0.26± 0.012	0.21 ± 0.012	0.18± 0.01
	Aquatic	BC	0.38± 0.017	0.31± 0.014	0.27± 0.01	0.39± 0.015	0.33± 0.013	0.29± 0.009
		TK	0.32± 0.018	0.25 ± 0.015	0.22± 0.011	0.33± 0.017	0.26 ± 0.015	0.23± 0.01
SSI (*mV*^2^)	Land	BC	266.33± 15.2	245.58± 11.61	221.81± 10.13	267.02± 14.8	246.08± 11.3	222.14± 9.8
		TK	221.42± 11.2	207.56± 9.4	196.36± 8.5	222.25± 10.91	207.89± 9.1	197.66± 7.9
	Aquatic	BC	321.42± 17.1	295.46± 13.4	277.25± 12.1	323.36± 16.9	295.59± 13.1	279.41± 11.9
		TK	261.19± 13.1	239.67± 12.9	221.56± 11.9	262.52± 12.9	239.81± 12.8	222.41± 11.5
RMS (*mV*)	Land	BC	0.41± 0.017	0.36± 0.013	0.33± 0.011	0.42± 0.015	0.37± 0.012	0.34± 0.01
		TK	0.33± 0.017	0.29 ± 0.015	0.27± 0.012	0.34± 0.016	0.30 ± 0.015	0.28± 0.01
	Aquatic	BC	0.47± 0.019	0.40± 0.016	0.37± 0.014	0.48± 0.018	0.41± 0.014	0.38± 0.013
		TK	0.40± 0.02	0.35 ± 0.017	0.32± 0.016	0.41± 0.019	0.36 ± 0.016	0.33± 0.016
AAC (*mV*)	Land	BC	0.069± 0.009	0.064± 0.008	0.059± 0.007	0.068± 0.008	0.064± 0.007	0.058± 0.006
		TK	0.056± 0.007	0.051 ± 0.007	0.049 ± 0.006	0.055± 0.006	0.05 ± 0.007	0.049 ± 0.006
	Aquatic	BC	0.085± 0.008	0.077± 0.007	0.072± 0.006	0.084± 0.007	0.076± 0.006	0.072± 0.006
		TK	0.067± 0.008	0.061 ± 0.006	0.055 ± 0.006	0.067± 0.007	0.060 ± 0.006	0.055 ± 0.006
VAR (*mV*^2^)	Land	BC	0.064± 0.005	0.058± 0.005	0.053± 0.004	0.064± 0.006	0.057± 0.005	0.051± 0.004
		TK	0.053± 0.006	0.047 ± 0.006	0.043 ± 0.004	0.054± 0.005	0.046 ± 0.006	0.042 ± 0.004
	Aquatic	BC	0.08± 0.005	0.073± 0.005	0.069± 0.005	0.08± 0.006	0.072± 0.005	0.069± 0.005
		TK	0.065± 0.006	0.059 ± 0.006	0.055 ± 0.005	0.064± 0.006	0.057 ± 0.006	0.054 ± 0.005
MNF (*Hz*)	Land	BC	62.34± 13	74.65± 14.6	82.45± 16.2	62.34± 13	72.85± 14.6	82.65± 16.24
		TK	55.34± 11.2	64.65± 14.2	75.45± 15.8	55.34± 11.2	63.15± 14.2	75.45± 15.8
	Aquatic	BC	85.34± 15.6	92.65± 17.6	101.45± 18.6	85.34± 15.6	92.65± 17.6	101.45± 18.6
		TK	77.34± 13.6	83.65± 14.2	92.45± 16.2	77.34± 13.6	83.65± 14.2	92.45± 16.2
MDF (*Hz*)	Land	BC	53.84± 9.8	66.12± 11.8	72.16± 13.72	52.99± 9.1	65.83± 11.2	71.01± 13.21
		TK	44.84± 9.6	56.12±11.8	63.16± 13.8	44.02± 8.9	55.72±11.1	62.86± 12.9
	Aquatic	BC	78.84± 12.4	89.12± 15.2	96.16± 16.2	78.02± 11.8	88.69± 14.7	95.73± 15.7
		TK	64.12± 10.4	72.3± 13	86.2± 14.2	63.94± 9.8	71.8± 12.7	85.8± 13.8
PKF (*Hz*)	Land	BC	53.84± 9.8	66.12± 11.8	72.16± 13.72	52.99± 9.1	65.83± 11.2	71.01± 13.21
		TK	44.84± 9.6	56.12±11.8	63.16± 13.8	44.02± 8.9	55.72±11.1	62.86± 12.9
	Aquatic	BC	78.84± 12.4	89.12± 15.2	96.16± 16.2	78.02± 11.8	88.69± 14.7	95.73± 15.7
		TK	64.12± 10.4	72.3± 13	86.2± 14.2	63.94± 9.8	71.8± 12.7	85.8± 13.8
mACC (*g*)	Land	BC	1.39± 0.14	1.75± 0.16	3.9± 0.26	1.39± 0.14	1.75± 0.16	3.9± 0.26
		TK	1.52± 0.18	1.93± 0.2	4.32± 0.33	1.52± 0.18	1.93± 0.2	4.32± 0.33
	Aquatic	BC	1.3± 0.1	1.65± 0.12	3.7± 0.2	1.3± 0.1	1.65± 0.12	3.7± 0.2
		TK	1.43± 0.12	1.81± 0.14	4.07± 0.24	1.43± 0.12	1.81± 0.14	4.07± 0.24
mGYR (°/*sec*)	Land	BC	21.32± 4.2	48.78± 8.6	95.12± 16.4	21.32± 4.2	48.78± 8.6	95.12± 16.4
		TK	23.46±5.1	53.66± 10.32	104.6± 19.8	23.46±5.1	53.66± 10.32	104.6± 19.8
	Aquatic	BC	19.2± 3.2	44± 7.2	85.6± 14.2	19.4± 3.17	44± 7.2	85.6± 14.2
		TK	23.03±4.16	52.7± 9.3	102.7± 18.4	23.03±4.16	52.7± 9.3	102.7± 18.4

## Data Availability

The raw data supporting the conclusions of this article will be made available by the authors, without undue reservation.
